# Prevalence of psychological violence in the employed Brazilian population and its occurrence in the workplace: National Survey of Health 2019

**DOI:** 10.1590/1980-549720250025

**Published:** 2025-06-02

**Authors:** Priscila Maria Stolses Bergamo Francisco, Daniela de Assumpção, Marcia Bandini, Sergio Roberto de Lucca

**Affiliations:** I Universidade Estadual de Campinas, School of Medical Sciences, Department of Public Health – Campinas (SP), Brazil.; II Universidade Estadual de Campinas, School of Medical Sciences, Graduate Program in Gerontology – Campinas (SP), Brazil.

**Keywords:** Workforce, Workplace violence, Adult health, Occupational health, Health surveys

## Abstract

**Objective:**

To estimate the prevalence of psychological violence among employed adults aged 18-69 years; to verify the distribution of sociodemographic and lifestyle characteristics within those who have reported psychological violence in the workplace; and to estimate the number of individuals who have reported this type of violence perpetrated by the employer/boss in the workplace.

**Methods:**

This is a descriptive study with data from the National Survey of Health (2019). Employed people were selected in relation to the workforce. Psychological violence was defined as follows: in the last 12 months, “has anyone offended, humiliated, or mocked you in front of other people?”. For those who reported violence, the location of the incident and who the aggressor was were verified. The prevalence of psychological violence was estimated and the characteristics of those who suffered this type of violence were described by absolute and relative frequencies.

**Results:**

The prevalence of psychological violence reached 11% (95% confidence interval [CI]: 10.5–11.6) of the employed adult population, was higher among women, individuals between 18–29 years old, Black people, single, and among those with income up to 0.5 minimum wage. Of the individuals who suffered psychological violence in the workplace, 57.6% were men, 31.9% were between 30–39 years old, 47% were mixed-race, 44.4% completed high school, 39.8% had an income between one and two minimum wages, 13.5% were smokers, 37.6% consumed alcoholic beverages, and 23.8% consumed alcohol excessively. As for the aggressor, 28.8% (around two million people) reported that psychological violence in the workplace was committed by the employer/boss.

**Conclusion:**

According to the study results, among the employed Brazilian population, 11% reported psychological violence in the last year; we identified the segments most affected by psychological violence in the workplace and measured this violence having the boss/employer as the aggressor.

## INTRODUCTION

According to the World Health Organization (WHO), workplace violence results from a complex interaction between several factors, especially the work conditions and organization, as well as worker-aggressor interaction^1^. It is characterized by incidents that include offense, abuse, threat, or attack in working circumstances and whose impact affects the health of workers^2^. As per recent data from the International Labour Organization (ILO, 2022), violence and moral and sexual harassment still affect 738 million workers in the work environment^3^. People who have suffered discrimination by gender, race/nationality/ethnicity, skin color, religion, or disability were the most affected^3^.

The identification of psychological violence can be difficult, particularly when there are no physical consequences related to it. However, authors of several studies point to the negative impacts on the psychological and physical health of workers, affecting work performance and, sometimes, leading professionals to abandon work^4,5,6,7^. In a cross-sectional study carried out on salaried employees, mostly from the private sector, of the three main metropolitan areas of Chile, psychological distress reached 30.8% in women and 16.5% in men classified as vulnerable in the workplace, according to the instrument adopted by the study^8^. Authors of a systematic review and meta-analysis of 253 studies (1987 to 2018), whose objective was to investigate the prevalence and factors associated with workplace violence in healthcare professionals, identified that 42.5% of the participants reported exposure to violence, mainly verbal abuse (57.6%), threats (33.2%), and sexual harassment (12.4%)^9^. Authors of another systematic review and meta-analysis, which included 41 studies on workplace violence among nurses from 13 countries, found a prevalence of 58% for various types of violence, including verbal abuse (64%), threat (30%), and bullying or mobbing — psychological harassment (25%)^10^.

In the Brazilian literature, research on the topic is relatively scarce, and in most studies participants are from the health care and education sectors^7^. According to data from an integrative review of 20 articles published between 2006 and 2016, overall, there is a consensus that moral harassment in the workplace is a violence that involves suffering, abuse of power, verbal aggression, humiliation, discrimination, with serious consequences in the professional and family life and in the health of workers, including manifestations of stress, low self-esteem and confidence, destabilization, fragility, and psychic and/or physical damage^7^. It should be noted that the impact of psychological violence in the workplace has mobilized the union representations as a discussion agenda in collective negotiations^11^.

The publication of the ILO Convention No. 190 (C190) (2019) on the eradication of moral and gender harassment rekindled the relevance and importance of the debate among member countries; in Brazil, C190 is in the process of ratification^12^. Within this context, in this study, our objective was to estimate the prevalence of psychological violence among adults aged 18 to 69 years; to verify the distribution of sociodemographic and lifestyle characteristics concerning those who reported psychological violence in the workplace; and to estimate the number of individuals who reported psychological violence perpetrated by the employer/boss in the workplace.

## METHODS

In the present study, a subsample of data from the National Survey of Health (*Pesquisa Nacional de Saúde* – PNS 2019) was considered, composed exclusively of individuals aged 18 to 69 years (n=78,358), classified as employed in the reference week adopted by the survey (July 21 to 27, 2019; n=50,896), who suffered psychological violence in the last year (n=5,604), and who reported having suffered this violence at the workplace (n=1,606). PNS is the largest household health survey ever conducted in Brazil, whose aim is to provide information on the determinants, conditioning factors, and health needs of the Brazilian population, including exposure to violence, for formulating public policies and making effective health interventions^13^. PNS microdata are available for public access and use at the research’s website (https://www.pns.icict.fiocruz.br/) by clicking on: *Sobre a pesquisa* [About the research]*; Bases de dados (PNS 2019, Microdados IBGE)* [Databases (PNS 2019, IBGE Microdata)]*; Arquivos de Microdados da PNS 2019* [Microdata Archives of PNS 2019].

The PNS sample was obtained by probabilistic cluster sampling procedures, in three stages: census tracts or set of census tracts, households, and residents, having one resident drawn from each household selected to answer the specific questionnaire. In the three stages, the draws were carried out by simple random sampling. Questions about violence were answered only by residents aged ≥18 years^13^. Details on the questionnaires and methods of PNS 2019 are published elsewhere^13,14^.

Regarding the position in the workforce, employed people were considered those who, in the reference week of the survey, worked at least one full hour in paid work or in work without direct compensation — providing support to the economic activity of a household member or relative — or, still, those who had paid work from which they were temporarily absent this week, as defined by the survey^14^.

**Figure 1.**
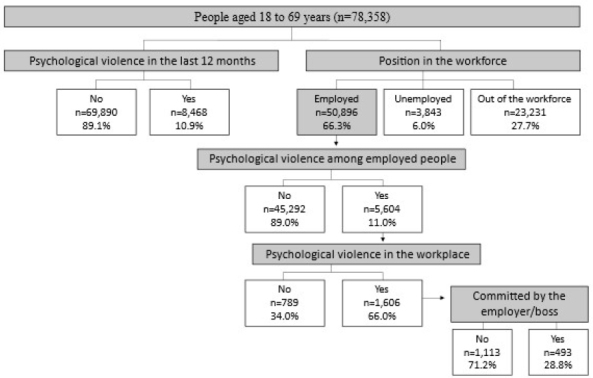
Flow for creating the subsample used in the present study (n=78,358). National Survey of Health, 2019.

Psychological violence in the 12 months prior to the interview was investigated through the question: *“Has anyone offended you, humiliated, or mocked you in front of other people?”* (yes; no). People who answered “yes” were asked about the place of occurrence by the question *“Where did this happen?”*, whose response options were: residence; workplace; school, college, or other educational establishment; bar or restaurant; public highway or other public place; the Internet, social media, or mobile phone; other. There were also questions about the aggressor: *“Who did that to you?”*, with the category of interest of the study, “employer/boss,” being one of the options of response, in addition to the others (spouse or partner; ex-spouse or ex-partner; partner, boyfriend/girlfriend, ex-partner, ex-boyfriend/girlfriend; father, mother, stepfather or stepmother; son/daughter, stepson/stepdaughter; brother/sister; other relative; friend/colleague/neighbor; coworker in general; unknown person; police officer; other). In Figure 1, the steps for creating the subsample analyzed in this study are shown. The following variables evaluated by PNS were considered:

*Psychological violence* (yes; no): Among those who answered “yes,” information was obtained about the place of occurrence and who the aggressor was.

*Socio-demographic characteristics*: sex (men; women), age group (18 to 29; 30 to 39; 40 to 49; 50 to 69 years), race/skin color (white; Black; mixed-race), marital status (with spouse; without spouse), level of education (some elementary school; elementary school; high school; college degree), and per capita household income in minimum wages (≤0.5; >0.5 and ≤1.0; >1.0 and ≤2.0; >2.0).

*Health behaviors:* alcohol consumption defined by the usual frequency of once or more per month (yes; no), alcohol abuse characterized by the consumption of five doses or more on a single occasion in the last 30 days (yes; no), and current tobacco smoker (yes; no).

The percentages correspond to the estimates for proportions that considered the sample weights of the research.

Initially, the prevalence of psychological violence in the employed population was verified, according to sociodemographic characteristics and health behaviors; the differences were verified by Pearson’s χ^2^ test (Rao & Scott), considering a 5% significance level. Among the people who suffered psychological violence, the place of occurrence was verified and, for those who reported “at the workplace,” the description of the characteristics was performed by means of percentage relative frequencies and respective 95% confidence intervals (95%CI), according to the selected variables.

For the population estimate of people who suffered psychological violence committed by the employer/boss, the population projection of the subgroup aged 18 to 69 years (141,462,885 individuals) made available by the Brazilian Institute of Geography and Statistics (IBGE) for the year 2019^15^ was considered, and the percentages obtained from the present study were applied (Figure 1). The analyses were carried out in the Stata software version 15.0, in the *survey* module, which takes into account the sample and post-stratification weights.

The PNS project was approved by the National Commission of Ethics in Research with Human Beings of the Brazilian Ministry of Health (Process No. 3.529.376, of August 23, 2019). Participation in the survey was voluntary and all respondents signed an informed consent form.

## RESULTS

In 2019, the prevalence of psychological violence in the employed population aged 18 to 69 years reached 11% and was significantly higher among women, younger individuals (18 to 29 years), who self-reported to be Black and mixed-race, in relation to whites, among those who were single, who smoked, and who consumed alcohol excessively. Conversely, the prevalence of psychological violence was lower among individuals with per capita household income higher than two minimum wages, compared to the subgroup with income ≤0.5 minimum wage (Table 1).

**Table 1.** Characteristics of the employed population (18 to 69 years) and prevalence of psychological violence in the last 12 months, according to sociodemographic and lifestyle variables (n=50,896). National Survey of Health, 2019.

Variables	n (%)	Prevalence (95%CI)	p-value*
Sex			
Men	28,668 (55.4)	9.7 (9.0–10.4)	p<0.001
Women	22,228 (44.6)	12.7 (11.8–13.5)	
Total	50,896	11.0 (10.5–11.6)	
Age (years)			
18 to 29	9,744 (23.7)	13.7 (12.4–15.2)	p<0.001
30 to 39	14,047 (27.6)	10.8 (9.7–12.1)	
40 to 49	12,650 (23.2)	10.4 (9.4–11.5)	
50 to 69	14,455 (25.5)	9.2 (8.3–10.1)	
Race/skin color			
White	18,548 (43.9)	9.8 (9.1–10.7)	p<0.001
Black	6,068 (11.9)	12.2 (10.8–13.7)	
Mixed-race	25,497 (42.8)	11.9 (11.1–12.8)	
Asian/Indigenous	776 (1.4)	10.8 (7.6–15.1)	
Marital status			
With spouse	20,610 (44.8)	9.0 (8.3–9.8)	p<0.001
Without spouse	30,286 (55.2)	12.6 (11.8–13.4)	
Level of education			
Some elementary school	15,171 (24.9)	11.4 (10.4–12.6)	p=0.491
Elementary school	7,187 (14.6)	11.7 (10.4–13.1)	
High school	18,259 (40.0)	10.8 (9.9–11.6)	
College degree	10,279 (20.5)	10.5 (9.3–11.9)	
Per capita household income (MW)**			
≤0.5	10,722 (16.8)	13.0 (11.7–14.4)	p=0.002
>0.5 and ≤1.0	13,655 (27.7)	11.0 (10.2–12.0)	
>1.0 and ≤2.0	14,391 (31.7)	10.9 (9.8–12.1)	
>2.0	12,107 (23.8)	9.7 (8.8–10.7)	
Current smoker			
No	44,042 (87.0)	10.4 (9.8–11.0)	p<0.001
Yes	6,854 (13.0)	15.0 (13.6–16.6)	
Alcoholic beverage intake (≥1 time/month)			
No	32,881 (62.3)	10.7 (10.0–11.6)	p=0.241
Yes	18,015 (37.7)	11.5 (10.6–12.3)	
Abusive alcohol consumption			
No	39,213 (77.5)	10.4 (9.8–11.1)	p<0.001
Yes	11,683 (22.5)	13.0 (11.9–14.2)	

95%CI: 95% confidence interval;

^*^p-value of Pearson’s χ^2^ test (Rao & Scott);

^**^MW: Minimum wage.

**Figure 2.**
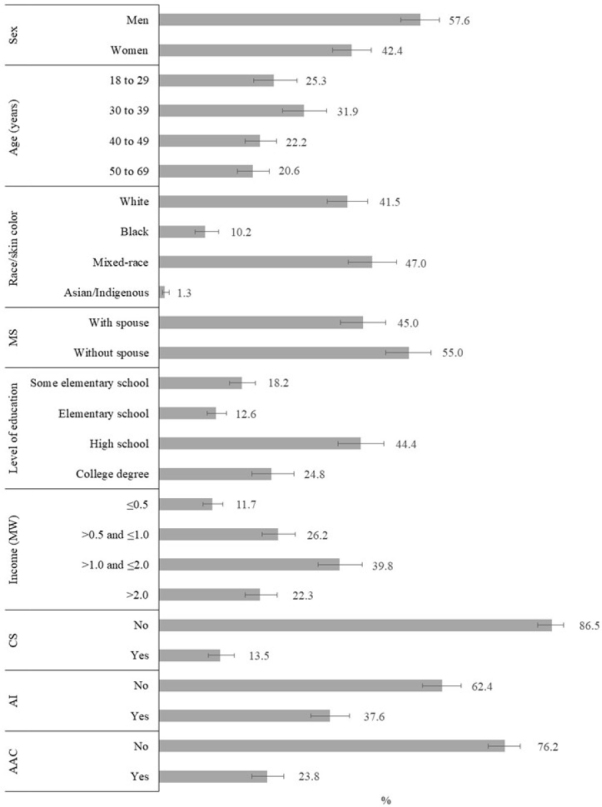
Distribution of individuals aged 18 to 69 years who suffered psychological violence in the last 12 months in the workplace (n=1,606). National Survey of Health, 2019.

MS: Marital status; Income (MW): per capita household income in minimum wages; CS: current smoker; AI: alcohol intake (≥1 time/month); AAC: abusive alcohol consumption.

Among the people employed in the reference week of the survey who reported psychological violence in the last 12 months, 28.8% reported the occurrence at the workplace, whose mean age was 38.6 years (95%CI 37.6–39.6), and the distribution of sociodemographic characteristics and health behaviors are shown in Figure 2. We observed a higher proportion of men (57.6%), people aged 30 to 39 years (31.9%) compared to those aged 40 years or older, mixed-race (47.0%), with high school level of education (44.4%), and per capita household income between one and two minimum wages (39.8%). Regarding lifestyle, we found that 13.5% smoked, 37.6% consumed alcoholic beverages (≥1 time/month), and 23.8% consumed alcohol excessively.

Considering the individuals who reported psychological violence in the workplace, regarding the aggressor, 28.8% (95%CI 25.0–32.8) mentioned that the violence was committed by the employer/boss, representing approximately two million people (data not presented in tables).

## DISCUSSION

According to the study results, the prevalence of psychological violence among the employed Brazilian population was 11%, and the sociodemographic profile was similar to that observed in the study conducted by Mascarenhas et al.^16^, who considered the Brazilian adult population (≥18 years). There was also a higher occurrence among those with lower income, smokers, and those who consumed alcohol excessively. As pointed out by Vieira^17^, violence, illness, and health at work are not insensitive to gender, race, and class, among other social markers of inequality. Authors of a systematic review with 215 articles published between 2005 and 2015, which considered the results of occupational surveys conducted in Brazil, identified that most of the studies were from the health care and education sectors; in addition, only three studies addressed the issue of violence^18^, which indicates the need for research on the subject.

In this study, among people aged 18 to 69 years who reported psychological violence in the workplace in the last 12 months, regarding the sociodemographic profile, men, young adults, mixed-race skin color, people with high school level of education, and with income between one and two minimum wages were those who most frequently experienced some episode of violence at the workplace, according to data of the PNS 2019. Based on the study “Labor Survey of the Labor Direction” (*Encuesta Laboral de la Dirección del Trabajo* – Encla, 2019), held in Chile, 10.1% of formal workers witnessed some violence in the workplace (moral/sexual harassment, public disparagement/mocking)^19^. It should be noted that we did not find studies whose authors have characterized the sociodemographic profile of adults who suffered psychological violence at the workplace.

Work is an important social determination of health in general and especially mental health. Several occupational stressors related to the organization of work are known, such as the overburden and imbalance in the division of tasks and power, which can give rise to moral harassment, among other types of violence, described by the WHO since 1986. The strong association between violence in the workplace and the impacts on workers’ mental and physical health is recognized^20^.

Violence is a social phenomenon that permeates Brazilian society and manifests itself in the form of physical and/or psychological aggression. While physical violence is an important predictor of mortality from external causes (homicides, work-related accidents, traffic accidents), psychological violence compromises the mental health of its victims and may trigger mental and behavioral disorders, suicidal behavior, and suicides^11,21^. According to the National Policy on the Reduction of Morbimortality by Accidents and Violence (*Política Nacional de Redução da Morbimortalidade por Acidentes e Violências*), of the Ministry of Health, violence is defined as *an event represented by actions performed by individuals, groups, classes, nations, which cause physical, emotional, moral and/or spiritual damage to oneself or others*, and psychological violence is one of the forms of expression of this aggravation^22^. Regardless of the place of occurrence, its impact on the population represents a serious public health issue and, when this type of violence occurs in the workplace, it causes negative impacts on the physical and mental health of workers^22,23^.

The relationship between violence and increased risk of suicide has also been pointed out by several studies. In France, psychological violence perpetrated by the hierarchical superiors in the workplace was widely debated after the occurrence of serial suicides by workers of a telephone company^24^. Severe stress at work, fear of losing employment, harassment and psychosocial factors at work (PFW) increase the risk of and attempted suicide in workers^25^.

In Brazil, PFW are recognized by the Ministry of Health as risk factors for mental illness and also for work-related musculoskeletal disorders, according to the updated version of the List of Work-related Diseases (*Lista de Doenças Relacionadas ao Trabalho* – LDRT), published in 2023, with emphasis on physical or psychological violence related to labor aspects^26^. The National Policy on Occupational Health (*Política Nacional de Saúde do Trabalhador e da Trabalhadora*)^27^ provides for the adoption of actions based on the recognition of work-related violence. The identification of these forms of violence *may equip the production of knowledge for supporting the elaboration of more accurate diagnoses on the condition of the Brazilian worker* ^27^.

The emotional suffering in its various forms of manifestation is of compulsory notification. These manifestations may include: easily crying, sadness, excessive fear, psychosomatic disorders, agitation, irritation, nervousness, anxiety, tachycardia, sweating, insecurity, among other symptoms, which may indicate the development or aggravation of mental disorders, as per the International Classification of Diseases (ICD-10): mental and behavioral disorders (F00 to F99); alcoholism (Y90 and Y91); burnout syndrome (Z73.0); symptoms and signs related to cognition, perception, emotional state, and behavior (R40 to R46); people with potential health risks related to socioeconomic and psychosocial circumstances (Z55 to Z65); circumstances related to working conditions (Y96); and intentional self-harm (X60 to X84), which have as their causal elements work-related risk factors, whether resulting from its organization and management or from the exposure to certain toxic agents^28^.

Nonetheless, the invisibility of psychological violence in the workplace is evident, as well as the historical underreporting of work-related diseases and illnesses from the perspective of mental health. From 2006 to 2017, 8,474 cases of work-related mental disorders throughout Brazil were recorded in the Notifiable Diseases Information System (*Sistema de Informação de Agravos de Notificação* – SINAN)^29^. Researchers, such as Pintor and Garbin^30^, seek ways to combat this invisibility of work-related violence through workers’ health surveillance.

Our findings contribute to increasing the knowledge about psychological violence manifested in the work environment among the employed adult Brazilian population, evidencing this phenomenon according to sociodemographic and lifestyle characteristics. However, in addition to the data, it should be noted that, in view of the complexity of this phenomenon, other approaches are necessary to better understand the causes of violence. The concept of intersectionality, as an analytical tool, enables the approach of more than one form of simultaneous oppression. Thus, discriminatory processes are no longer understood separately to take into account their complexity, seeking to identify the specific conditions arising from them^31^. It should be noted that intersectionality allows for a more accurate perception of violence, particularly that related to work, and contributes to a better understanding of labor health and illness processes^17^.

Although most of the psychological violence in the workplace was not committed by the employer/boss, about two million people reported this type of violence. It is worth noting that, in the workplace, the approaches of the phenomenon in the individual sphere — harasser/victim — are predominant and denote that the abuse of psychological power over the victim produces a wrenching effect. In work-related violence, there is a convergence of markers of social inequality that, in power relations, can (re)produce and accentuate social stereotypes and generate processes of humiliation and exclusion^17^.

In this study, we verified that 32.5 and 24.2% of psychological violence in the workplace (data not presented) were committed by friends/colleagues and unknown people, respectively, suggesting that violence is a widespread social phenomenon and is present in various relationships in the workplace, that is, more and more human relations seem to generate aggressions in the workplace^5,30,32,33^.

Moreover, it is worth mentioning that hostile acts, although individually performed, have as a background the organization of work, corporate culture, leadership style, and are influenced by competition and competitiveness, driven by the process of economic globalization. Thus, institutional or organizational harassment is the result of harassing management practices that take place sequentially and predominantly vertically, from top to bottom^32-34^.

Not by chance, several initiatives have highlighted the importance of adopting measures to prevent violence and harassment in the workplace. In 2020, the ILO published a guideline on safe and healthy working environments free from violence and harassment, which highlights the importance of acting on psychosocial factors triggering labor stress, as well as forms of discrimination, such as ethnicity, gender, place of origin, so as to avoid social stereotypes, especially workers in situations of vulnerability^11^.

In 2022, the WHO published a guideline with evidence-based recommendations on interventions that can be implemented in the workplace to prevent, protect, promote, and support workers’ mental health^35^. In Brazil, the Ministry of Labor updated the Regulatory Standard No. 5, including topics related to the prevention and fight against sexual harassment and other types of violence in the workplace in its activities and practices, in 2023^36^. Occupational psychosocial stressors, such as violence that manifests in the workplace and is often presented in healthcare services, including emergency ones, show that something must be done in prevention. As Leka^37^ argues, instead of looking at the situation as an additional problem or calamity, with skepticism or fear, giving visibility to the scandalous cases of psychological violence in the workplace may be an opportunity for individuals, organizations, and society in general.

However, some limitations should be mentioned. We measured psychological violence by a single question concerning the occurrence of offense, humiliation, or mocking in front of other people, not by an instrument or a set of questions that would allow us to measure other forms of violence of this nature. The question had as reference the previous 12 months, so it is possible that memory bias compromises the report of psychological violence, either by forgetfulness or by social naturalization of the phenomenon in people’s daily lives. Furthermore, the employed population was defined according to an indicator proposed by the ILO, adopted by IBGE, and not based on a question that would identify psychological violence by type of performed work. Regarding psychosocial factors at work, we could not conclude that individual behaviors (smoking and alcohol consumption) have a cause-and-effect relationship with the occurrence of violence in the workplace. Conversely, this is a study with a representative coverage of the Brazilian adult population, with standardized research procedures, which obtained information collected in households, making the report about violence in the workplace more reliable. In addition, it is noteworthy that the use of the indicator “position in the workforce” provides a more complete picture of the different forms of work, and not only of professionals with formal employment.

In this study, we showed that, among the employed Brazilian population, 11% reported psychological violence in the last year; identified the segments most affected by this type of violence in the workplace; and dimensioned this violence having the employer/boss as the aggressor. Among those who suffered psychological violence in the workplace, most were men, people aged 30 to 39 years, mixed-race, with high school level of education, and per capita household income between one and two minimum wages. The research also allowed us to measure that 28.8% of psychological violence (about two million people) was committed by the employer/boss at the workplace. The promotion of healthy work environments, with relationships founded on respect, solidarity, compassion, and kindness, is a challenging task, but possible, although nowadays violence and hatred are banalized.
