# Supplementary material for: Prevalence of psychological violence in the employed Brazilian population and its occurrence in the workplace: National Survey of Health 2019
Source: Rev Bras Epidemiol. 2025 Jun 2;28:e250025. doi: 10.1590/1980-549720250025 (PMC12129241; doi:10.1590/1980-549720250025)
Supplement: Translation

# Translation: Prevalência de violência psicológica na população brasileira ocupada e sua ocorrência no ambiente de trabalho: Pesquisa Nacional de Saúde 2019

**DOI:** 10.1590/1980-549720250025.2

**Authors:** Priscila Maria Stolses Bergamo Francisco, Daniela de Assumpção, Marcia Bandini, Sergio Roberto de Lucca

**Affiliations:** I Universidade Estadual de Campinas, Faculdade de Ciências Médicas, Departamento de Saúde Coletiva – Campinas (SP), Brasil.; II Universidade Estadual de Campinas, Faculdade de Ciências Médicas, Programa de Pós-Graduação em Gerontologia – Campinas (SP), Brasil.

**Keywords:** Força de trabalho, Violência no trabalho, Saúde do adulto, Saúde do Trabalhador, Inquéritos epidemiológicos

## Abstract

**Objetivo:**

Estimar a prevalência de violência psicológica (VP) entre adultos ocupados com 18-69 anos; verificar a distribuição das características sociodemográficas e de estilo de vida naqueles que referiram VP no ambiente de trabalho; e estimar o número de indivíduos que referiu VP perpetrada por patrão/patroa/chefe no trabalho.

**Métodos:**

Estudo descritivo com dados da Pesquisa Nacional de Saúde (2019). Selecionaram-se pessoas ocupadas com relação à força de trabalho. A VP foi definida assim: nos últimos 12 meses, “*alguém te ofendeu, humilhou ou ridicularizou na frente de outras pessoas?*”. Para os que referiram violência, verificaram-se o local da ocorrência e quem foi o agressor. Estimou-se a prevalência da VP e foram descritas, por meio de frequências absolutas e relativas, as características dos que sofreram VP.

**Resultados:**

A prevalência de VP atingiu 11% (intervalo de confiança de 95% — IC95%10,5–11,6) na população adulta ocupada, foi maior nas mulheres, naqueles de 18–29 anos, nos pretos e pardos, sem cônjuge, com renda de até 0,5 salário-mínimo. Dos indivíduos que sofreram VP no local de trabalho, 57,6% eram homens, 31,9% tinham 30–39 anos, 47% eram pardos, 44,4% tinham ensino médio completo, 39,8% renda entre 1 e 2 salários-mínimos, 13,5% fumavam, 37,6% ingeriam bebidas alcoólicas e 23,8% consumiam álcool abusivamente. Quanto ao agressor, 28,8% (cerca de 2 milhões de pessoas) referiram que a VP no ambiente laboral foi cometida por patrão/patroa/chefe.

**Conclusão:**

O estudo mostrou que, na população brasileira ocupada, 11% referiu VP no último ano, identificou os segmentos mais afetados pela VP no trabalho e dimensionou essa violência tendo patrão/patroa/chefe como agressor.

## INTRODUÇÃO

Para a Organização Mundial da Saúde (OMS), a violência no trabalho resulta de uma interação complexa de vários fatores, especialmente as condições e a organização do trabalho, assim como a interação trabalhador-agressor^1^. Caracteriza-se por incidentes que compreendem ofensa, abuso, ameaça ou ataque em circunstâncias de trabalho e cujo impacto afeta a saúde dos trabalhadores^2^. Dados recentes da Organização Internacional do Trabalho (OIT, 2022) revelam que a violência e o assédio moral e sexual ainda afetam 738 milhões de trabalhadores e trabalhadoras no mundo do trabalho^3^. As pessoas que sofreram discriminação por gênero, raça/nacionalidade/etnia, cor da pele, religião ou alguma incapacidade foram as mais atingidas^3^.

A identificação da violência psicológica pode ser difícil, particularmente quando não se veem consequências físicas a ela relacionada. Contudo, diversos estudos apontam para os impactos negativos na saúde psíquica e física dos trabalhadores e das trabalhadoras, afetando o desempenho laboral e, por vezes, levando o profissional ao abandono do trabalho^4,5,6,7^. Em estudo transversal realizado com trabalhadores assalariados, majoritariamente do setor privado, das três principais áreas metropolitanas do Chile, o sofrimento psicológico alcançou 30,8% nas mulheres e 16,5% nos homens classificados em condição de vulnerabilidade no local de trabalho, conforme o instrumento adotado no estudo^8^. Uma revisão sistemática e metanálise de 253 estudos (1987 a 2018), cujo objetivo foi investigar a prevalência e fatores associados à violência no local de trabalho em profissionais da saúde, identificou que 42,5% dos participantes relataram exposição à violência, principalmente abuso verbal (57,6%), ameaças (33,2%) e assédio sexual (12,4%)^9^. Outra revisão sistemática e metanálise que incluiu 41 estudos e que explorou a violência no trabalho entre enfermeiras de 13 países encontrou prevalência de 58% para diversas formas de violência, entre elas abuso verbal (64%), ameaça (30%) e *bullying* ou *mobbing* — assédio psicológico (25%)^10^.

Na literatura nacional, as pesquisas sobre o tema são relativamente escassas, e na maioria dos estudos os participantes são dos setores saúde e educação^7^. Dados de uma revisão integrativa com 20 artigos publicados entre 2006 e 2016 mostram que, de maneira geral, há um consenso de que o assédio moral no trabalho é uma violência que envolve sofrimentos, abusos de poder, agressões verbais, humilhações, discriminações, com graves consequências na vida profissional e familiar e na saúde dos trabalhadores e trabalhadoras, incluindo manifestações de estresse, baixa da autoestima e da confiança, desestabilização, fragilização e danos psíquicos e/ou físicos^7^. Cabe ressaltar que o impacto da violência psicológica no trabalho tem mobilizado as representações sindicais como pauta de discussão nas negociações coletivas^11^.

A publicação da Convenção 190 da OIT (2019) de eliminação do assédio moral e de gênero reacendeu a relevância e a importância do debate entre os países membros, e no Brasil a C190 se encontra em processo de ratificação^12^. Nesse contexto, o objetivo do presente estudo foi estimar a prevalência de violência psicológica entre adultos ocupados com 18 a 69 anos de idade; verificar a distribuição das características sociodemográficas e de estilo de vida naqueles que referiram violência psicológica no ambiente de trabalho; e estimar o número de indivíduos que referiu violência psicológica perpetrada por patrão/chefe no trabalho.

## MÉTODOS

O presente estudo considerou uma subamostra dos dados da Pesquisa Nacional de Saúde (PNS 2019), composta exclusivamente de indivíduos com 18 a 69 anos de idade (n=78.358) classificados como ocupados na semana de referência adotada pela pesquisa (21 a 27 de julho de 2019; n=50.896) que sofreram violência psicológica no último ano (n=5.604) e que reportaram ter sofrido essa violência no ambiente de trabalho (n=1.606). A PNS é o maior inquérito domiciliar de saúde já realizado no Brasil e tem por objetivo prover informações sobre os determinantes, condicionantes e necessidades de saúde da população brasileira, inclusive a exposição à violência, para a formulação de políticas públicas e o alcance de intervenções efetivas em saúde^13^. Os microdados da PNS encontram-se disponíveis para acesso e uso público no endereço eletrônico da pesquisa (https://www.pns.icict.fiocruz.br/) por meio do caminho: *Sobre a pesquisa; Bases de dados (PNS 2019, Microdados IBGE); Arquivos de Microdados da PNS 2019*.

A amostra da PNS foi obtida por procedimentos de amostragem probabilística por conglomerados, em três estágios: setores censitários ou conjunto de setores, domicílios e moradores, sendo sorteado um morador de cada domicílio selecionado para responder ao questionário específico. Nos três estágios, os sorteios foram realizados por amostragem aleatória simples. As questões sobre violência foram respondidas apenas pelos moradores com idade ≥ 18 anos^13^. Detalhes sobre os questionários e os métodos da PNS 2019 encontram-se publicados^13,14^.

Com relação à posição na força de trabalho, foi considerada como ocupada a pessoa que, na semana de referência da pesquisa, trabalhou pelo menos uma hora completa em trabalho remunerado ou em trabalho sem remuneração direta — em ajuda à atividade econômica de membro do domicílio ou parente — ou, ainda, a que tinha trabalho remunerado do qual estava temporariamente afastada nessa semana, conforme definição adotada pela pesquisa^14^.

A violência psicológica nos 12 meses anteriores à entrevista foi investigada por meio da pergunta: *“Alguém te ofendeu, humilhou ou ridicularizou na frente de outras pessoas?”* (sim; não). As pessoas que responderam “sim” foram questionadas sobre o local de ocorrência pela pergunta “*Onde isso ocorreu?”*, cujas opções de resposta eram: residência; trabalho; escola, faculdade ou outro estabelecimento de ensino; bar ou restaurante; via pública ou outro local público; internet, redes sociais ou celular; outro. Também foi inquirido sobre o agressor: *“Quem fez isso com você?”*, com a categoria de interesse do estudo, “patrão/patroa ou chefe”, sendo uma das opções de resposta, além das demais (cônjuge ou companheiro/a; ex-cônjuge ou ex-companheiro/a; parceiro/a, namorado/a, ex-parceiro/a, ex-namorado/a; pai, mãe, padrasto ou madrasta; filho/a, enteado/a; irmão/a; outro parente; amigo/a/colega, vizinho/a; empregado/a em geral; pessoa desconhecida; policial; outro). A Figura 1 informa sobre os passos da construção da subamostra analisada neste estudo. Foram consideradas as seguintes variáveis avaliadas pela PNS:

*Violência psicológica* (sim; não). Entre os que responderam “sim”, foram obtidas informações sobre o local de ocorrência e quem foi o agressor.

*Sociodemográficas:* sexo (masculino; feminino), faixa etária (18 a 29; 30 a 39; 40 a 49; 50 a 69 anos), raça/cor da pele (branca; preta; parda), estado conjugal (com cônjuge; sem cônjuge), escolaridade (ensino fundamental incompleto; fundamental completo; médio completo; superior completo) e renda domiciliar *per capita* em salários-mínimos (≤ 0,5; > 0,5 e ≤ 1,0; > 1,0 e ≤ 2,0; > 2,0).

*Comportamentos de saúde:* consumo de álcool definido pela frequência habitual de uma vez ou mais por mês (sim; não), uso abusivo de álcool caracterizado pelo consumo de cinco doses ou mais em uma única ocasião nos últimos 30 dias (sim; não) e fumante atual de tabaco (sim; não).

**Figura 1.** Fluxo da construção da subamostra utilizada neste estudo (n=78.358). Pesquisa Nacional de Saúde, 2019.

Os percentuais correspondem às estimativas para proporções que levaram em conta os pesos amostrais da pesquisa.

Inicialmente, foi verificada a prevalência de violência psicológica na população ocupada, segundo características sociodemográficas e de comportamentos de saúde; as diferenças foram verificadas por meio do teste qui-quadrado de Pearson (Rao & Scott), considerando-se o nível de significância de 5%. Entre as pessoas que sofreram violência psicológica, foi verificado o local da ocorrência e, para aquelas que referiram “no trabalho”, foi realizada a descrição das características por meio de frequências relativas percentuais e respectivos intervalos de confiança de 95% (IC95%), de acordo com as variáveis selecionadas.

Para a estimativa populacional das pessoas que sofreram violência psicológica cometida por patrão/patroa ou chefe, partiu-se da projeção populacional do subgrupo com 18 a 69 anos (141.462.885 de indivíduos) disponibilizada pelo Instituto Brasileiro de Geografia e Estatística (IBGE) para o ano de 2019^15^, e foram aplicados os percentuais obtidos no presente estudo (Figura 1). As análises foram realizadas no *software* Stata versão 15.0, no módulo *survey*, que leva em conta os pesos amostrais e de pós-estratificação.

O projeto da PNS foi aprovado na Comissão Nacional de Ética em Pesquisa para Seres Humanos do Ministério da Saúde (Processo nº 3.529.376, de 23 de agosto de 2019). A participação na pesquisa foi voluntária e todos os entrevistados assinaram um termo de consentimento livre e esclarecido.

## RESULTADOS

Em 2019, a prevalência de violência psicológica na população ocupada, com 18 a 69 anos de idade, alcançou 11% e foi significativamente maior nas mulheres, nos indivíduos mais jovens (18 a 29 anos), que se autodeclararam pretos e pardos, em relação aos brancos, nos que não tinham cônjuge, que fumavam e que usavam bebida alcoólica de forma abusiva. Por outro lado, a prevalência de violência psicológica foi menor entre os indivíduos com renda domiciliar *per capita* superior a dois salários-mínimos, em comparação ao subgrupo com renda ≤ 0,5 salário mínimo (Tabela 1).

**Tabela 1.** Características da população ocupada (18 a 69 anos) e prevalência da violência psicológica nos últimos 12 meses, segundo variáveis sociodemográficas e de estilo de vida (n= 50.896). Pesquisa Nacional de Saúde, 2019.

Variáveis	n (%)	Prevalência (IC95%)	Valor de p*
Sexo			
Masculino	28.668 (55,4)	9,7 (9,0–10,4)	p<0,001
Feminino	22.228 (44,6)	12,7 (11,8–13,5)	
Total	50.896	11,0 (10,5–11,6)	
Idade (em anos)			
18 a 29	9.744 (23,7)	13,7 (12,4–15,2)	p<0,001
30 a 39	14.047 (27,6)	10,8 (9,7–12,1)	
40 a 49	12.650 (23,2)	10,4 (9,4–11,5)	
50 a 69	14.455 (25,5)	9,2 (8,3–10,1)	
Raça/cor da pele			
Branca	18.548 (43,9)	9,8 (9,1–10,7)	p<0,001
Preta	6.068 (11,9)	12,2 (10,8–13,7)	
Parda	25.497 (42,8)	11,9 (11,1–12,8)	
Amarela/indígena	776 (1,4)	10,8 (7,6–15,1)	
Estado conjugal			
Com cônjuge	20.610 (44,8)	9,0 (8,3–9,8)	p<0,001
Sem cônjuge	30.286 (55,2)	12,6 (11,8–13,4)	
Escolaridade			
Fundamental incompleto	15.171 (24,9)	11,4 (10,4–12,6)	p=0,491
Fundamental completo	7.187 (14,6)	11,7 (10,4–13,1)	
Médio completo	18.259 (40,0)	10,8 (9,9–11,6)	
Superior completo	10.279 (20,5)	10,5 (9,3–11,9)	
Renda domiciliar per capita (SM)**			
≤ 0,5	10.722 (16,8)	13,0 (11,7–14,4)	p=0,002
> 0,5 e ≤ 1,0	13.655 (27,7)	11,0 (10,2–12,0)	
> 1,0 e ≤ 2,0	14.391 (31,7)	10,9 (9,8–12,1)	
> 2,0	12.107 (23,8)	9,7 (8,8–10,7)	
Fumante atual			
Não	44.042 (87,0)	10,4 (9,8–11,0)	p<0,001
Sim	6.854 (13,0)	15,0 (13,6–16,6)	
Ingestão de bebida alcoólica (≥1 vez/mês)			
Não	32.881 (62,3)	10,7 (10,0–11,6)	p=0,241
Sim	18.015 (37,7)	11,5 (10,6–12,3)	
Consumo abusivo de bebida alcoólica			
Não	39.213 (77,5)	10,4 (9,8–11,1)	p<0,001
Sim	11.683 (22,5)	13,0 (11,9–14,2)	

IC95%: intervalo de confiança de 95%.

^*^ Valor de p do teste qui-quadrado de Pearson (Rao & Scott).

^**^ SM: salário-mínimo.

Entre as pessoas ocupadas na semana de referência da pesquisa que referiram violência psicológica nos últimos 12 meses, 28,8% relataram a ocorrência no local de trabalho, cuja média de idade foi de 38,6 anos (IC95% 37,6–39,6) e a distribuição das características sociodemográficas e de comportamentos de saúde estão apresentadas na Figura 2. Observou-se maior proporção de homens (57,6%), de pessoas com 30 a 39 anos (31,9%) em comparação às de 40 anos ou mais, pardos (47,0%), com ensino médio completo (44,4%) e com renda domiciliar *per capita* entre 1 e 2 salários-mínimos (39,8%). Com relação ao estilo de vida, verificou-se que 13,5% fumavam, 37,6% ingeriam bebidas alcoólicas (≥ 1 vez/mês) e 23,8% consumiam álcool de forma abusiva.

**Figura 2.** Distribuição de indivíduos com 18 a 69 anos que sofreram violência psicológica nos últimos 12 meses, no ambiente de trabalho (n=1.606). Pesquisa Nacional de Saúde, 2019.

EC: estado conjugal; Renda (SM): renda domiciliar *per capita* em salário-mínimo; FA: fumante atual; IA: ingestão de álcool (≥1 vez/mês); CAA: consumo abusivo de álcool.

Considerando-se os indivíduos que relataram a violência psicológica no ambiente laboral, quanto ao agressor, 28,8% (IC95% 25,0–32,8) mencionaram que a violência foi cometida por patrão/patroa ou chefe, representando aproximadamente 2 milhões de pessoas (dado não apresentado em tabela).

## DISCUSSÃO

Os resultados do estudo mostram que a prevalência de violência psicológica entre a população brasileira ocupada foi de 11%, e o perfil sociodemográfico foi semelhante ao observado no estudo de Mascarenhas et al.^16^, que considerou a população adulta brasileira (≥ 18 anos). Também foi verificada maior ocorrência entre aqueles com menor renda, nos fumantes e nos que consumiam bebidas alcoólicas abusivamente. Como apontado por Vieira^17^, a violência, o adoecimento e a saúde no trabalho não são insensíveis ao gênero, à raça e à classe, entre outros marcadores sociais de desigualdade. Uma revisão sistemática com 215 artigos publicados entre 2005 e 2015, que considerou resultados de inquéritos ocupacionais conduzidos no Brasil, identificou que a maioria das produções eram do ramo de saúde e educação; além disso, somente três estudos abordaram o tema da violência^18^, o que denota a necessidade de estudos nessa temática.

Neste estudo, entre as pessoas com 18 a 69 anos que relataram violência psicológica no ambiente laboral nos últimos 12 meses, quanto ao perfil sociodemográfico, homens, adultos jovens, pardos, pessoas com nível médio de ensino e com renda entre 1 e 2 salários-mínimos foram os que mais frequentemente vivenciaram algum episódio de violência no trabalho, de acordo com os dados da PNS de 2019. Com base na *Encuesta Laboral de la Dirección del Trabajo* (Encla, 2019), realizada no Chile, 10,1% dos trabalhadores formais presenciaram alguma violência no local de trabalho (assédio moral/sexual, depreciação/ridicularização pública)^19^. Cabe ressaltar que não foram encontrados na literatura estudos que tenham caracterizado o perfil sociodemográfico de adultos que sofreram violência psicológica no trabalho.

O trabalho é um importante determinante social da saúde em geral e especialmente da saúde mental. São conhecidos vários estressores ocupacionais relacionados à organização do trabalho, como a sobrecarga e o desequilíbrio na divisão de tarefas e poder, que podem dar lugar ao assédio moral, entre outras formas de violência, descritas pela OMS desde 1986. É reconhecida a forte associação entre violência no trabalho e os impactos sobre a saúde mental e física de trabalhadores^20^.

A violência é um fenômeno social que perpassa pela sociedade brasileira e se manifesta na forma de agressão física e/ou psicológica. Enquanto a violência física é importante preditor de mortalidade por causa externa (homicídios, acidentes de trabalho, acidentes de trânsito), a violência psicológica compromete a saúde mental de suas vítimas e pode desencadear transtornos mentais e comportamentais, comportamento suicida e suicídios^11,21^. Segundo a Política Nacional de Redução da Morbimortalidade por Acidentes e Violências, do Ministério da Saúde, a violência é definida como *evento representado por ações realizadas por indivíduos, grupos, classes, nações, que ocasionam danos físicos, emocionais, morais e/ou espirituais a si próprio ou a outros*, e a violência psicológica é uma das formas de expressão desse agravo^22^. Independentemente do local de ocorrência, o seu impacto na população representa um grave problema de saúde pública e, quando essa forma de violência ocorre no ambiente de trabalho, causa impactos negativos na saúde física e mental dos trabalhadores^22,23^.

A relação entre violência e aumento do risco de suicídio também tem sido apontada por vários estudos. Na França, a violência psicológica perpetrada pelos superiores hierárquicos nos ambientes de trabalho foi amplamente debatida após a ocorrência de suicídios em série, de trabalhadores de uma empresa de telefonia^24^. Estresse grave no trabalho, medo de perder o emprego, assédio e fatores psicossociais no trabalho (FPT) aumentam o risco e tentativa de suicídio nos trabalhadores^25^.

No Brasil, os FPT são reconhecidos pelo Ministério da Saúde como fatores de risco para o adoecimento mental e também para os distúrbios osteomusculares relacionados ao trabalho, conforme versão atualizada da Lista de Doenças Relacionadas ao Trabalho (LDRT), publicada em 2023, com destaque para a violência física ou psicológica relacionada a aspectos laborais^26^. A Política Nacional de Saúde do Trabalhador e da Trabalhadora^27^ prevê a adoção de ações baseadas no reconhecimento das violências relacionadas ao trabalho. A identificação dessas violências *poderá instrumentalizar a produção de conhecimentos que fundamentem a elaboração de diagnósticos mais precisos sobre a condição do trabalhador brasileiro* ^27^.

É de notificação compulsória também o caso de sofrimento emocional em suas diversas formas de manifestação, tais como: choro fácil, tristeza, medo excessivo, doenças psicossomáticas, agitação, irritação, nervosismo, ansiedade, taquicardia, sudorese, insegurança, entre outros sintomas, que podem indicar o desenvolvimento ou agravo de transtornos mentais, utilizando a Classificação Internacional de Doenças (CID-10): transtornos mentais e comportamentais (F00 a F99); alcoolismo (Y90 e Y91); síndrome de *burnout* (Z73.0); sintomas e sinais relativos à cognição, à percepção, ao estado emocional e ao comportamento (R40 a R46); pessoas com riscos potenciais à saúde relacionados com circunstâncias socioeconômicas e psicossociais (Z55 a Z65); circunstância relativa às condições de trabalho (Y96); e lesão autoprovocada intencionalmente (X60 a X84), os quais têm como elementos causais fatores de risco relacionados ao trabalho, sejam eles resultantes da sua organização e gestão, seja por exposição a determinados agentes tóxicos^28^.

Ainda assim, é evidente a invisibilidade da violência psicológica no trabalho, bem como a subnotificação histórica das doenças e agravos relacionados ao trabalho na perspectiva da saúde mental. No período de 2006 a 2017, foram registrados no Sistema de Informação de Agravos de Notificação (SINAN) 8.474 casos de transtornos mentais relacionados ao trabalho em todo o Brasil^29^. Estudos como o de Pintor e Garbin^30^ buscam formas de combater essa invisibilização das violências relacionadas ao trabalho por meio de vigilância em saúde do trabalhador.

Nossos achados contribuem para ampliar o conhecimento sobre a violência psicológica manifestada no ambiente laboral entre a população adulta brasileira ocupada, desvelando esse fenômeno segundo características sociodemográficas e de estilo de vida. No entanto, para além dos dados, deve-se considerar que diante da complexidade desse fenômeno outras abordagens tornam-se necessárias para melhor compreensão das causas da violência. O conceito de interseccionalidade, enquanto ferramenta analítica, possibilita a abordagem de mais de uma forma de opressão simultânea. Desse modo, os processos discriminatórios deixam de ser compreendidos isoladamente para levar em conta a sua complexidade, buscando-se identificar as condições específicas que deles decorrem^31^. Cabe ressaltar que a interseccionalidade possibilita uma percepção mais acurada da violência, particularmente aquela relacionada ao trabalho, e contribui para melhor entendimento dos processos de saúde e de adoecimento laboral^17^.

Embora a maior parte da violência psicológica no local de trabalho não tenha sido cometida por patrão/patroa ou chefe, cerca de 2 milhões de pessoas relataram esta violência. Deve-se considerar que, nos ambientes de trabalho, as abordagens do fenômeno no campo individual — assediador/vítima — são predominantes e denotam que o abuso de poder psicológico sobre a vítima produz um efeito desolador. Nas violências relacionadas ao trabalho, observa-se uma convergência de marcadores de desigualdade social que, nas relações de poder, podem (re)produzir e acentuar estereótipos sociais e gerar processos de humilhação e exclusão^17^.

Neste estudo foi verificado que 32,5 e 24,2% da violência psicológica no ambiente de trabalho (dados não apresentados) foi cometida por amigo/colega e pessoa desconhecida, respectivamente, sugerindo que a violência é um fenômeno social disseminado e presente em diversas relações no local de trabalho, ou seja, cada vez mais as relações humanas parecem gerar agressões no espaço laboral^5,30,32,33^.

Além disso, pontua-se que os atos hostis, ainda que individualmente praticados, têm como pano de fundo a organização do trabalho, a cultura corporativa, o estilo de liderança, e são influenciados pela concorrência e competitividade, impulsionados pelo processo de globalização econômica. Desse modo, o assédio institucional ou organizacional é resultante de práticas de gestão assediadoras que ocorrem em cascata e predominantemente de forma vertical, de cima para baixo^32,33,34^.

Não por acaso, várias iniciativas têm destacado a importância da adoção de medidas de prevenção da violência e do assédio no ambiente de trabalho. Em 2020, a OIT publicou diretriz sobre ambientes de trabalho seguros e saudáveis livres de violência e de assédio, que destaca a importância da atuação sobre os fatores psicossociais desencadeantes de estresse laboral, bem como as formas de discriminação, tais como etnia, sexo, gênero, local de procedência, para que se evitem estereótipos sociais, especialmente os trabalhadores e as trabalhadoras em situações de vulnerabilidade^11^.

Em 2022, a OMS publicou diretriz com recomendações baseadas em evidências sobre intervenções que podem ser implementadas nos locais de trabalho para prevenir, proteger, promover e apoiar a saúde mental dos trabalhadores^35^. No Brasil, o Ministério do Trabalho atualizou a Norma Regulamentadora nº 5, incluindo temas referentes à prevenção e ao combate ao assédio sexual e a outras formas de violência no trabalho nas suas atividades e práticas, em 2023^36^. Psicoestressores ocupacionais, como a violência que explode nos ambientes de trabalho e se apresenta frequentemente nos serviços de saúde, incluindo os de emergência, demonstram que algo precisa ser feito na prevenção. Como defende Leka^37^, em vez de encarar a situação como um problema adicional ou uma calamidade, com ceticismo ou medo, dar visibilidade aos escandalosos casos de violência psicológica no trabalho talvez seja uma oportunidade para os indivíduos, as organizações e a sociedade em geral.

Entretanto, algumas limitações precisam ser mencionadas. A violência psicológica foi medida por uma única pergunta sobre a ocorrência de ofensa, humilhação ou ridicularização na frente de outras pessoas, e não por um instrumento ou um conjunto de questões que permitiriam mensurar outras formas de violência dessa natureza. A pergunta utilizada teve como referência os 12 meses anteriores, portanto é possível que o viés de memória comprometa o relato da violência psicológica, seja por esquecimento, seja por naturalização social do fenômeno no cotidiano das pessoas. Também, a definição de população ocupada provém de um indicador proposto pela OIT, adotado pelo IBGE, e não de uma pergunta que permitisse identificar a violência psicológica por tipo de trabalho desempenhado. No que se refere aos fatores psicossociais no trabalho, não se pode concluir que os comportamentos individuais (tabagismo e consumo de álcool) tenham relação de causa e efeito com a ocorrência da violência no local de trabalho. Em contrapartida, trata-se de um estudo com abrangência representativa da população adulta do Brasil, com procedimentos de pesquisa padronizados, que obteve informações coletadas nos domicílios, tornando o relato sobre violência no ambiente de trabalho mais fidedigno. Além disso, deve-se considerar que o uso do indicador da condição na força de trabalho propicia um quadro mais completo das distintas formas de trabalho, e não só de profissionais com vínculo formal empregatício.

O estudo mostrou que, na população brasileira ocupada, 11% referiram violência psicológica no último ano, identificou os segmentos mais afetados pela VP no trabalho e dimensionou essa violência tendo patrão/patroa/chefe como agressor. Entre os que sofreram violência psicológica no local de trabalho, a maioria era composta de homens, por pessoas com 30 a 39 anos, pardos, com ensino médio completo e renda domiciliar *per capita* entre 1 e 2 salários-mínimos. A pesquisa também permitiu mensurar que 28,8% da violência psicológica (cerca de 2 milhões de pessoas) foi cometida por patrão/patroa ou chefe no ambiente de trabalho. A promoção de ambientes de trabalho saudáveis, com relações fundadas no respeito, solidariedade, compaixão e gentileza, é tarefa desafiadora, mas possível, apesar de os tempos de hoje serem pautados pela banalização da violência e do ódio.
